# Evaluation of Multifarious Plant Growth Promoting Trials of Yeast Isolated from the Soil of Assam Tea (*Camellia sinensis* var. *assamica*) Plantations in Northern Thailand

**DOI:** 10.3390/microorganisms8081168

**Published:** 2020-08-01

**Authors:** Jaturong Kumla, Supakorn Nundaeng, Nakarin Suwannarach, Saisamorn Lumyong

**Affiliations:** 1 Research Center of Microbial Diversity and Sustainable Utilization, Chiang Mai University, Chiang Mai 50200, Thailand; suwan.462@gmail.com (N.S.); scboi009@gmail.com (S.L.); 2 Department of Biology, Faculty of Science, Chiang Mai University, Chiang Mai 50200, Thailand; Supakorn.ning@gmail.com; 3 Academy of Science, The Royal Society of Thailand, Bangkok 10300, Thailand

**Keywords:** soil yeast, plant growth promoting, Assam tea plant, Thailand

## Abstract

Some soil microorganisms, especially bacteria and mycorrhizal fungi, play a role in the promotion of plant growth. However, plant growth promotion involving yeasts in soil has not yet been extensively investigated. This study aimed to isolate and identify yeast strains obtained from soils of the Assam tea plant (*Camellia sinensis* var. *assamica*) in northern Thailand and to investigate their plant growth promoting capabilities. A total of 42 yeast strains were obtained and identified by analysis of the D1/D2 domain of the large subunit ribosomal RNA gene. We identified 35 strains of six species belonging to the phylum Ascomycota, namely *Aureobasidium melanogenum, Kazachstania aquatica, Saturnispora diversa*, *Saturnispora sekii*, *Schwanniomyces pseudopolymorphus* and *Wickerhamomyces anomalus,* and six species were determined to belong to the phylum Basidiomycota, namely *Apiotrichum scarabaeorum, Curvibasidium pallidicorallinum*, *Papiliotrema laurentii*, *Rhodosporidiobolus ruineniae*, *Trichosporon asahii* and *Trichosporon coremiiforme*. Seven strains were representative of potential new species and belonged to the genera Galactomyces and Wickerhamomyces. A total of 28 strains were found to produce indole-3-acetic acid (IAA) in a range of 2.12 to 37.32 mg/L, with the highest amount of IAA produced by *R*. *ruineniae* SDBR-CMU-S1-03. All yeast strains were positive in terms of ammonia production, and only eight strains were positive for siderophore production. Two yeast species, *P. laurentii* and *W. anomalus*, were able to solubilize the insoluble form of calcium and zinc. The ability to produce amylase, endogulcanase, lipase, pectinase, protease and xylanase was dependent upon the yeast species and strain involved.

## 1. Introduction

The Assam tea plant (*Camellia sinensis* var. *assamica*) is a non-alcoholic caffeine-containing beverage crop that is primarily cultivated for its leaf [1,2]. This tea plant is native to East and Southeast Asia, but it is currently being cultivated worldwide in various tropical and subtropical regions [2,3]. The Assam tea plant is commonly grown in higher agricultural areas in northern Thailand at elevations of 450–1500 m above sea level. These areas include the provinces of Chiang Mai, Chiang Rai, Lampang, Mae Hong Son, Nan, Phayao and Phrae [4,5]. Normally, Assam tea leaves have been used to make black tea and “Miang”, a traditional unique fermented food of northern Thailand [5,6,7]. Soil degradation and the substantial quality of the tea have been associated with the long-term monoculture of tea plantations [8]. Chemical-based fertilizers are commonly used for the promotion of tea plant growth, which are also known to cause environmental pollution, soil acidification, heavy metal pollution, soil compaction and changes in soil microbial populations. These fertilizers are also known to be hazardous to the health of farmers as well [8,9,10]. Many research studies have been done on the effects of chemical fertilization in terms of both tea yield and quality [11,12,13]. For this reason, researchers have been interested in replacing chemical-based fertilizers with various techniques that involve the application of beneficial microorganisms, especially plant growth promoting microorganisms (PGPMs) that can support the sustainability of the agricultural industry and the environment [14,15,16].

PGPMs have been defined as microorganisms (fungi, bacteria, actinomycetes and yeasts) that can enhance plant growth, improve plant nutrient uptake, induce systematic resistance and increase stress tolerance through one of the following mechanisms nitrogen fixation, solubilization of insoluble minerals, exopolysaccharide secretion, biocontrol activity, phytohormone production, siderophores production etc. [17,18,19]. PGPMs are usually 1–2% of the total microorganisms that live in/on plants, the soil and the rhizosphere zone. These microorganisms have been categorized as free living and as having a symbiotic association with the plants [16,18]. PGPMs have been isolated from the endospheres, phyllospheres and rhizospheres of many plant species [20,21,22]. Yeasts are microorganisms that they are widely dispersed and commonly found on plant leaves, flowers, fruits and in the soil [23,24,25]. Some yeast species (*Aureobasidium pullulans, Candida tropicalis, Moesziomyces aphidis* and *Rhodotorula mucilaginosa*) have the ability to promote plant growth and have been identified as PGPMs [26,27,28,29]. Although, there have been relatively few studies on plant growth promoting yeasts obtained from soils. The objective of our study was to isolate the yeast strains obtained from the rhizosphere soil of Assam tea plantations located in northern Thailand, to identify them and to study their characteristics in terms of their plant growth promoting abilities (production of indole-3-acetic acid (IAA), siderophore, ammonia and extracellular enzymes, and the solubilization of insoluble minerals). The knowledge gained from this research study can be used to develop plant growth promoting yeast as a bioinoculant, which may replace the chemical-based fertilizers that are currently being used.

## 2. Materials and Methods

### 2.1. Sample Collection

**Figure 1:**
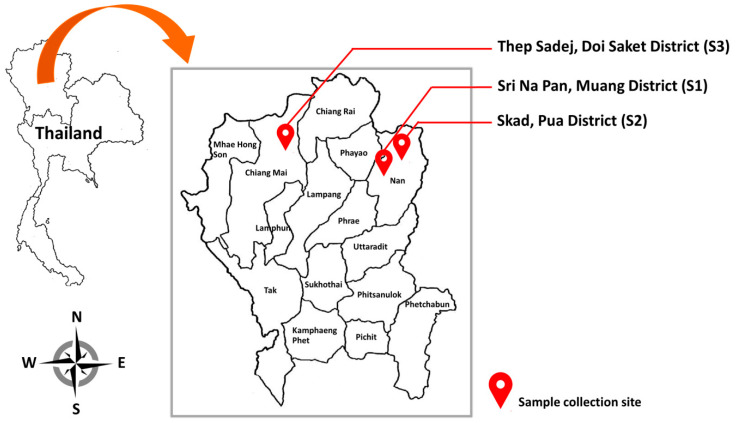
Map showing the location of sample collection sites.

Soil samples were collected from three Assam tea plantations (*Camellia sinensis* var. *assamica*) in Chiang Mai Province (Thep Sadej, Doi Saket District, 18°58’3” N 99°21’58” E), and Nan Province (Sri Na Pan, Muang District, 18°46’53” N 100°39’12” E and Skad, Pua District, 19°15’42” N 101°0’16” E), of northern Thailand during a one-year period from 2016 to 2017 (Figure 1). All collection sites were known to be monocultures of Assam tea. Rhizosphere soil samples of five plants were randomly collected at each site. Soil samples were aseptically collected at a depth of 5–10 cm from the surface soil in the rhizosphere zone using a stainless steel spade. The soil samples were kept in sterile plastic bags and carried to the laboratory on ice within 48 h of being collected. All collected soil samples were processed for the isolation of yeast immediately after reaching the laboratory. Type, pH value and electrical conductivity (EC) of the soil samples were determined at the Soil Science Laboratory, Faculty of Agriculture, Chiang Mai University, Thailand. The type, pH value and EC of the soil samples from each collection site are shown in Table 1.

### 2.2. Yeast Isolation

Yeast strains were isolated using the enrichment method with some modifications. We placed 1 gram of the soil sample in 99 mL of yeast malt extract (YM) broth (Sigma-Aldrich, St. Louis, MO, USA) that had been combined with 50 mg/L of chloramphenicol in 250 mL Erlenmeyer flasks. Flasks were placed on an orbital shaker with shaking at 150 rpm in the dark at 30 °C for 48 h. After 48 h of incubation, the dilution spread plate method was employed with seven serial dilutions in 0.5% NaCl solution. After dilution, 0.1 mL of suspension was spread on YM agar (Sigma-Aldrich, St. Louis, MO, USA) combined with 50 mg/L chloramphenicol. The plates were incubated at 30 °C for 48 h. Different yeast colonies were selected based on relevant morphological characteristics. The individual colonies were re-grown at 30 °C on YM agar for purification. The pure strains were kept in 20% glycerol at −80 °C and deposited in the Culture Collection of the Research Center of Microbial Diversity and Sustainable Utilization, Faculty of Science, Chiang Mai University, Thailand.

### 2.3. Yeast Identification

Each yeast strain was cultivated in 5 mL of YM broth in 18 × 180 mm test tubes with shaking at 150 rpm in the dark. After 48 h, yeast cells were harvested by centrifugation and washed three times with sterile distilled water. Genomic DNA was extracted using a Fungal DNA Extraction Mini Kit (FAVORGEN, Ping-Tung, Taiwan). The D1/D2 region of the large subunit ribosomal RNA (LSU rRNA) gene was amplified by polymerase chain reactions (PCR) using NL1 and NL4 primers [30]. The amplification process was performed in a polymerase chain reaction (PCR) that consisted of an initial denaturation at 95 °C for 5 min, followed by 35 cycles of denaturation at 95 °C for 30 s, annealing at 52 °C for 45 s, an extension at 72 °C for 1 min and 72 °C for 10 min. PCR products were checked and then purified using a NucleoSpin Gel and PCR Clean-up Kit (Macherey-Nagel, Germany). The purified PCR products were directly sequenced at the 1st Base Company (Kembangan, Malaysia). The obtained sequences were compared with type strain sequences deposited in the Genbank database via BLAST (http://blast.ncbi.nlm.nih.gov, 25 June 2020) for the purposes of species identification. For phylogenetic analysis, multiple sequence alignment was carried out using MUSCLE [31]. Phylogenetic trees were constructed using maximum likelihood by RAxML v. 7.0.3 under the GTR+I+G model with 1000 bootstrap replicates [32,33].

### 2.4. Determination of Indole-3-Acetic Acid (IAA) Production

Each yeast strain was grown in YM broth at 30 °C and shaken at 125 rpm for 48 h before being used. The cell density of each yeast strain was then adjusted using 0.5% NaCl solution relative to the 0.5 McFarland standard, which corresponded to 10^8^ CFU/mL. The resulting solution was then used as an inoculum. One hundred microliters of yeast inoculum were added to 10 mL of YM broth that had been supplemented with 2 mg/mL l-tryptophan (l-trp; Sigma-Aldrich, Steinheim, Germany). These specimens were held at a pH of 6.0 in 18 × 180 mm test tubes. Cultivation was performed in the dark at 30 °C with shaking at 150 rpm on an orbital shaker. After three days of incubation, the cultures were centrifuged at 11,000 rpm for 15 min to harvest the supernatant. The production of IAA of the tested strains was determined according to the colorimetric assay [28]. A pink to red color was considered positive for IAA production. Three replications were then made. The IAA in the supernatant was extracted and quantified with high performance liquid chromatography (HPLC) according to the method described by Kumla et al. [34].

### 2.5. Determination of Siderophore Production

The siderophore production was determined using chrome azurol S (CAS) agar [35]. One drop of yeast inoculum (5 µL of 0.5 McFarland standard) was dropped onto CAS agar. The inoculated plates were incubated at 30 °C in darkness for five days. The colonies producing a yellow, orange, purple or red zone were considered positive for siderophore production. Five replications were made.

### 2.6. Determination of Ammonia Production

A total of 100 microliters of yeast inoculum (0.5 McFarland standard) was added to 10 mL of peptone water at a pH of 6.0 in 18 × 180 mm test tubes. Cultivation was performed in the dark at 30 °C with shaking at 150 rpm on an orbital shaker. After 5 days of incubation, ammonia production was detected using Nessler’s reagent. The development of a yellow to brown color was indicative of a positive result for ammonia production [36]. Each treatment was performed in five replications.

### 2.7. Determination of Mineral Solubilization Ability

This experiment was carried out using basal medium (10.0 g glucose, 0.5 g (NH)_4_SO_4_, 0.2 g NaCl, 0.1 g MgSO_4_∙7H_2_O, 0.2 g KCl, 0.5 g yeast extract, 0.002g MnSO_4_∙H_2_O, and 15.0 g agar per liter of deionized water, pH 7.0) with the addition of non-soluble metal minerals including AlPO_4_, Ca_3_(PO_4_)_2_, CaCO_3_, CuCO_3_∙Cu(OH)_2_, CuO, CoCO_3_, FePO_4_, MgCO_3_, MnO, ZnCO_3_, ZnO, feldspar (KAlSi_3_O_8_), and kaolin (Al_2_Si_2_O_5_(OH)_4_) to a desired final concentration of 0.1% according to the method described by Fomina et al. [37]. One drop of yeast inoculum (5 µL of 0.5 McFarland standard) was dropped onto the tested agar. The inoculated plates were incubated at 30°C in darkness for 5 days. The colony diameter and solubilization zone (halo zone) were measured. Solubilization index (SI) was calculated as the halo zone diameter divided by the yeast colony diameter [38]. SI values of less than 1.0, between 1.0 and 2.0, and more than 2.0 were considered indicative of low, medium and high solubilization activities, respectively [37]. Five replications were made in each treatment.

### 2.8. Determination of Extracellular Enzyme Production

All yeast strains were investigated for extracellular enzyme production including endoglucanase, xylanase, pectinase, amylase, protease and lipase on agar plates. Enzyme production was reported as an enzyme activity index (EAI) and calculated as the ratio of the halo zone diameter and colony diameter [39]. One drop of yeast inoculum (5 µL of 0.5 McFarland standard) was dropped onto the tested agar. Five replications were made for each enzyme. Endoglucanase and xylanase productions were investigated on carboxymethyl cellulose (CMC) agar [40] and xylan agar [41,42], respectively. One drop of yeast inoculum (5 µL) was dropped onto the tested media. The inoculated media were incubated at 30 °C in darkness for five days. The yeast colonies were rinsed with sterile distilled water, and the enzyme activity was determined by staining with 1% Congo red and was then destained with 1 M NaCl for 15 min. The positive colonies produced a yellow zone of enzyme activity.

Pectinase and amylase productions were investigated on pectin agar [43] and starch agar [44], respectively. After inoculation, plates were incubated at 30 °C in darkness for 5 days. Pectinase and amylase activities were indicated by the development of a clear color change (yellow color) around or beneath the colonies after flooding the plate with 50 mM potassium iodide–iodine solution.

Protease production was tested on skim milk-YNB agar [45], and lipase production was determined on the Sierra’s agar [46]. After inoculation, plates were incubated at 30 °C in darkness for 5 days. The presence of a clear zone around or beneath the colony indicated enzyme activity.

## 3. Results

### 3.1. Yeast Isolation and Identification

**Figure 2:**
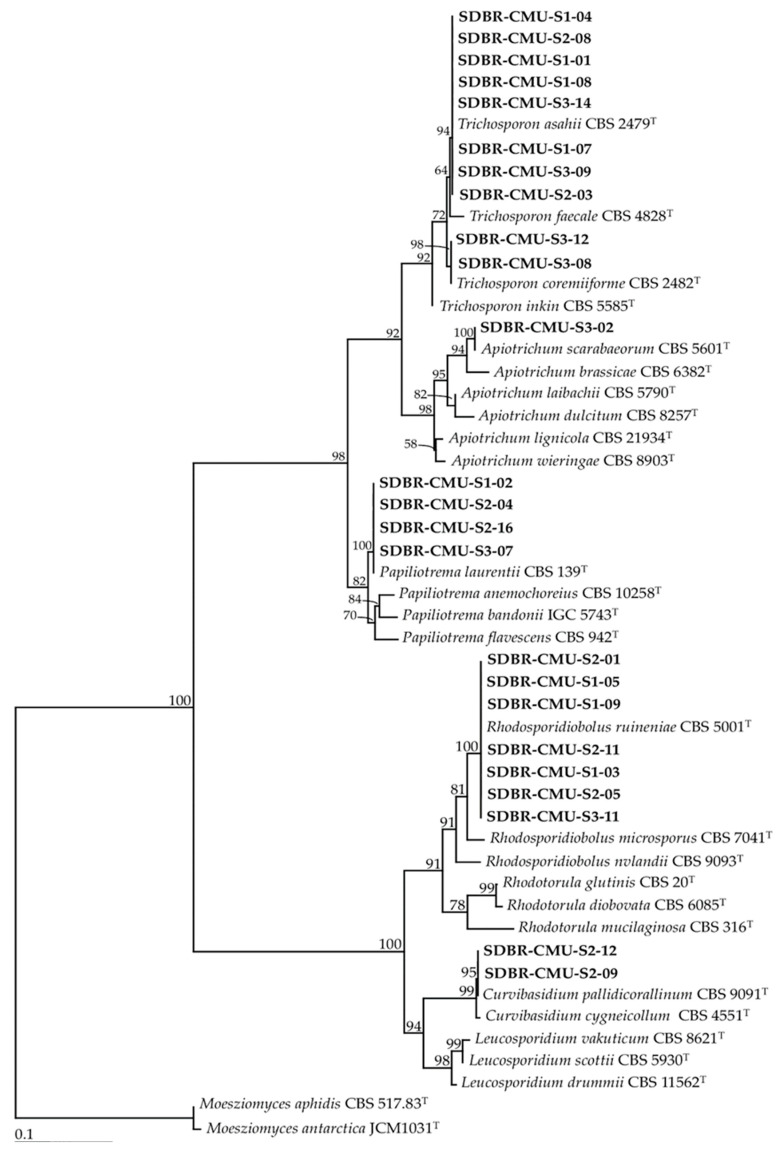
Phylogenetic tree of basidiomycetous yeast derived from maximum likelihood analysis of the D1/D2 region of the LSU rRNA gene. *Rhodotorula mucilaginosa* and *R. diobovata* were used as outgroup. Numbers above branches are the bootstrap statistics percentages. Branches with bootstrap values ≥ 50% are shown at each branch and the bar represent 0.1 substitutions per nucleotide position. The yeast strains obtained in this study are in bold. Superscription “T” means the type species.

**Figure 3:**
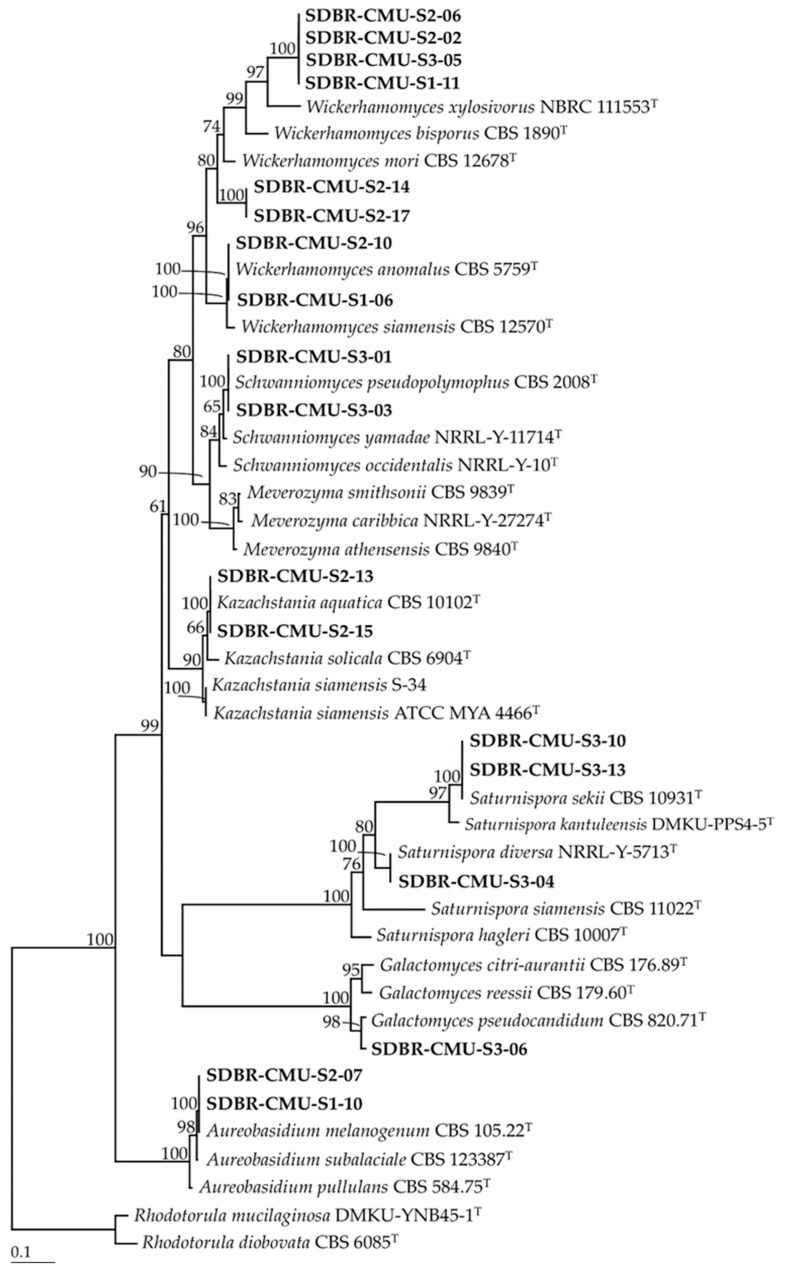
Phylogenetic tree of ascomycetous yeast derived from maximum likelihood analysis of the D1/D2 region of the LSU rRNA gene. *Moesziomyces antarctica* and *M. aphidis* were used as outgroup. Numbers above branches are the bootstrap statistics percentages. Branches with bootstrap values ≥ 50% are shown at each branch and the bar represent 0.1 substitutions per nucleotide position. The yeast strains obtained in this study are in bold. Superscription “T” means the type species.

The results revealed that 11, 17 and 14 strains of the isolated yeast strains were obtained from Site 1 (Skad, Pua District, Nan Province), Site 2 (Sri Na Pan, Muang District, Nan Province) and Site 3 (Thep Sadej, Doi Saket District, Chiang Mai Province), respectively. A total of 42 strains were identified as belonging to 12 known yeast species and three unrecognized species (Table 2, Figure 2 and Figure 3). It was found that 11 strains represented six species, namely *Aureobasidium melanogenum, Kazachstania aquatic, Saturnispora diversa, Saturnispora sekii, Schwanniomyces pseudopolymorphus*, and *Wickerhamomyces anomalus* belonged to the phylum Ascomycota. We identified 24 strains of six species, namely *Apiotrichum scarabaeorum, Curvibasidium pallidicorallinum, Papiliotrema laurentii, Rhodosporidiobolus ruineniae, Trichosporon asahii* and *Trichosporon coremiiforme* in the phylum Basidiomycota. Additionally, seven strains were categorized as previously unrecognized species belonging to the genera *Galactomyces* and *Wickerhamomyces*, and further study would be required for their identification.

### 3.2. Determination of IAA Production

Under culture conditions, all yeast strains were effectively able to grow. A total of 28 strains of six species (*Ap. scarabaeorum, C. pallidicorallinum, P. laurentii, R. ruineniae, T. asahii and T. coremiiforme* and one unrecognied species (*Wickerhamomyces* sp. 1) were positive for IAA production when tested by Salkowski’s reagent, as indicated by the formation of pink and red colors. The identification of IAA was confirmed by HPLC. The analysis indicated the presence of IAA produced from the yeast strains, which corresponded to the authentic IAA standard with a retention time of 10.1 min and a maximum absorption of 279 nm according to Kumla et al. [34]. The amount of IAA was also quantified by HPLC and is shown in Table 3. The amount of IAA ranged from 2.12 to 37.32 mg/L, which deepened according to the yeast strain. The highest amount of IAA was obtained from *R*. *ruineniae* SDBR-CMU-S1-03.

### 3.3. Determination of Siderophore and Ammonia Production

**Figure 4:**
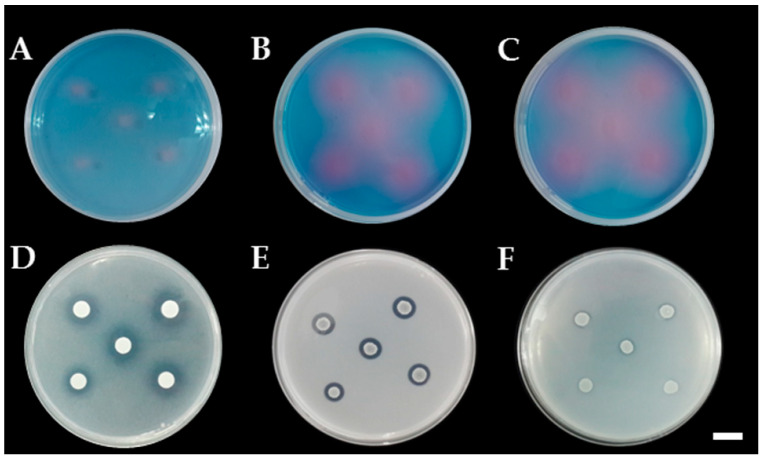
Color change zone on chrome azurol S (CAS) agar of siderophore production of *P. laurentii* SDBR-CMU-S1-02 (**A**), *Wickerhamomyces* sp. SDBR-CMU-S2-14 (**B**), and *Ap. scarabaeorum* SDBR-CMU-S3-02 (**C**). (**D**–**F**); clear zone of minerals solubilization, Ca_3_(PO_4_)_2_ solubilization by *W. anomalus* SDBR-CMU-S1-06 (**D**), ZnCO_3_ solubilization by *W. anomalus* SDBR-CMU-S1-06 (**E**), ZnO solubilization by *W. anomalus* SDBR-CMU-S1-06 (**F**). Scale bar = 10 mm.

Siderophore production was detected on CAS agar media. The results indicated that only eight yeast strains were positive for siderophore production by the formation of a pink zone around the colonies (Figure 4). These eight strains were identified to three known species that were named as *P*. *laurentii* (SDBR-CMU-S1-02, S2-04, S2-16 and S3-07), *A*. *melanogenum* (SDBR-CMU-S1-10) and *Ap*. *scarabaeorum* (SDBR-CMU-S3-02), and one recognized species named as *Wickerhamomyces* sp. 2 (SDBR-CMU-S2-14 and S2-17). In addition, all yeast strains were positive for ammonia production by the development of a yellow to brown color after Nessler’s reagent was added to each culture.

### 3.4. Determination of Mineral Solubilization Ability

The ability of all yeast strains to solubilize insoluble minerals was determined by producing a solubilization zone (clear zone) around the colony on the agar containing each insoluble mineral. The results indicated that all yeast strains were not able to produce a solubilization zone in all tested insoluble minerals, except for *P*. *laurentii* and *W*. *anomalus*. These strains belonging to two species were able to solubilize the insoluble form of phosphate and zinc (Table 4, Figure 4). Four strains of P. laurentii could solubilize Ca_3_(PO_4_)_2_ and ZnO at medium and high levels of activity, respectively. Two strains of W. anomalus displayed a medium degree of activity in terms of the solubilization of Ca_3_(PO_4_)_2_, ZnO and ZnCO_3_.

### 3.5. Determination of Extracellular Enzyme Production

**Figure 5:**
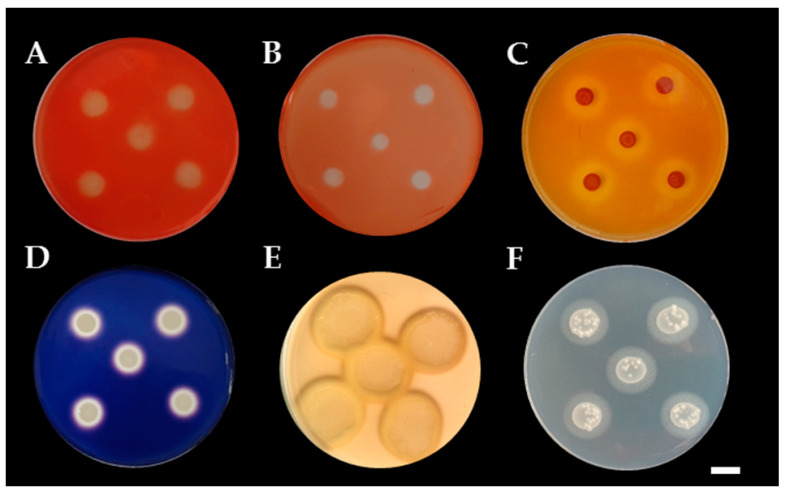
(**A**−**F**); Screening of extracellular enzyme production, endoglucanase production by *T. asahii* SDBR-CMU-S1-01 (**A**), xylanase production by *P. laurentii* SDBR-CMU-S1-02 (**B**), pectinase production *P. laurentii* SDBR-CMU-S1-02 (**C**), amylase production by *P. laurentii* SDBR-CMU-S2-04 (**D**), protease production by *W. anomalus* SDBR-CMU-S2-10 (**E**) and lipase production by *T. asahii* SDBR-CMU-S1-07 (**F**). Scale bar = 10 mm.

The ability of all yeast strains to produce extracellular enzymes was determined and the enzyme activity index is shown in Table 5 and Figure 5. The results indicate that the eight yeast strains of *K. aquatica, P. laurentii and S. pseudopolymorphus* produced amylase. Additionally, 23 strains belonging to five species (*R. ruineniae, S. pseudopolymorphus, T. asahii, T. coremiiforme and W. anomalus*) could produce endoglucanase. Lipase was produced by the 23 yeast strains of *R. ruineniae, P. laurentii, T. asahii, T. coremiiforme* and *Sat. diversa*. Pectinase production was observed in four of the strains of *P. laurentii*. Protease was produced by two strains of *W. anomalus* and four strains of *P. laurentii*. Lastly, 15 strains of *A. melanogenum, R. ruineniae, P. laurentii* and *Ap. scarabaeorum*, and one unrecognized species identified as *Galactomyces* sp., could produce xylanase.

## 4. Discussion

Soil is the most favorable environment for microorganisms including yeast strains [24,25]. In this study, both ascomycetous and basidiomycetous yeasts were recovered from the soil samples of the Assam tea plant (*C. sinensis* var. *assamica*) that had been collected in northern Thailand using the enrichment isolation technique. These results were in accordance with the findings of previous studies which reported that ascomycetous and basidiomycetous yeast strains were recovered from various soil samples [24,47,48,49]. Many ascomycetous yeast strains in the genera *Aureobasidium*, *Barnettozyma*, *Cyberlindnera*, *Kazachstania*, *Lipomyces* and *Schwanniomyces* are known to be generally more abundant and are more frequently found in soil [24,50,51]. *Apiotrichum*, *Cryptococcus*, *Papiliotrema*, *Rhodotorula* and *Trichosporon* were also identified as the dominant form of basidiomycetous yeast in soil samples [24,50,52]. In addition, yeast communities in the soil are influenced by both abiotic and biotic factors including soil organic matter content, pH, conductivity, temperature and the availability of water and macronutrients [52,53,54]. Among the 10 species identified in this study, *A. melanogenum, P. laurentii, R. ruineniae, S. pseudopolymorphus, Sat. diversa, Sat. sekii, T. asahii, T. coremiiforme, Ap. scarabaeorum* and *W. anomalus* are known to have been isolated from soil [28,52,53,55,56,57]. Interestingly, seven strains in the genera *Galactomyces* and *Wickerhamomyces* that were obtained in this study are representative of new potential species and will require further study.

In this study, certain plant growth promoting capabilities (the production of IAA, siderophore, ammonia and extracellular enzymes, and the solubilization of insoluble minerals) of all isolated yeast strains were investigated. IAA is a dominant type of auxin found in plants and is involved in certain specific plant growth responses identified as cell elongation, cell division, cell differentiation and root initiation [58,59]. The main precursor for IAA synthesis was l-trp [60]. IAA has been produced not only in plants, but also by certain microorganisms [60,61]. l-trp is present in root exudates and influences IAA production in microorganisms through only the Trp-dependent IAA biosynthetic pathway [62,63]. Previous studies have reported that yeast can produce IAA in the presence and absence of l-trp [26,27,64,65]. Our results revealed that 28 strains of six species (*Ap. scarabaeorum C. pallidicorallinum, P. laurentii, R. ruineniae, T. asahii and T. coremiiforme*) and one unrecognied species (*Wickerhamomyces* sp. 1) could produce IAA in liquid medium supplemented with l-trp with a supplemented amount of IAA ranging from 2.12 to 37.32 mg/L. Our findings agreed with the results of a number of previous studies that found that the amount of IAA production from microorganisms (including bacteria, yeast and fungi) was less than 100 mg/L [66,67,68]. However, the amount of IAA production obtained from yeast strains varied with different yeast species, and higher amounts of IAA production have been detected in some yeast strains. For example, Limtong et al. [69] found that *Rhodosporidium fluviale* DMKU-RK253 and *Candida michaelii* DMKU-RK343 could produce IAA at 565.1 and 259.0 mg/L, respectively. Additionally, *Rhodotorula paludigena* DMKU-RP301 showed the highest IAA production at 364.1 mg/L [65]. Fu et al. [26] found that *A. pullulans* JYC104 and *Rh. paludigena* JYC100 produced IAA at 610.63 and 400.59 mg/L, respectively. Moreover, several previous studies demonstrated that IAA-producing yeast strains could increase the root length of plants [23,26,64,70].

Most microorganisms produce siderophores as chelating agents for ferric ion. This not only supports plant growth but also indirectly inhibits plant pathogens by scavenging the available iron from the host environment [71,72,73]. In this study, *A. melanogenum*, *P. laurentii*, *Ap. scarabaeorum* and *Wickerhamomyces* sp. 2 were found to be positive for siderophore production. This result was supported by the findings of several previous studies which reported that some species of yeast in the genera Aureobasidium, Candida, Debaryomyces, Hanseniaspora, Holtermanniella, Lachancea, Meyerozyma, Pichia, Rhodotorula, Trichosporon and Wickerhamomyces could produce siderophores [64,65,74]. Ammonia production is one of the general characteristics of PGPMs [75]. Microbial production of ammonia potentially plays an important role in plant growth promotion via the availability of nitrogen [75,76]. The process of providing ammonia from microorganisms to the plant has been considered of vital importance. However, whether ammonia production is a common trait of yeast strains is unknown. In our study, all yeast strains possess ammonia-producing abilities. This result was similar to that of Fu et al. [26] who found that some yeast strains, namely *A. pullulans*, *Galactomyces candidum*, *Hanseniaspora uvarum*, *Kazachstania jiainica*, *Meyerozyma caribbica*, *M. aphidis, P. laurentii* and *R. ruineniae*, isolated from the rhizospheres of *Drosera spatulata* could produce ammonia.

Mineral-solubilizing microorganisms play a role in utilizing the available nutrition by enhancing their availability to plants through the solubilization and mineralization in the soil [77,78]. Mineral solubilization is influenced by the combined effect of a decrease in pH value and a decrease in organic acid production [77,78,79]. In this study, we evaluated the mineral-solubilizing ability of isolated yeast strains. The results indicated that only two yeast species, *P. laurentii* and *W. anomalus*, were able to solubilize the insoluble form of phosphate (Ca_3_(PO_4_)_2_), and zinc (ZnO and ZnCO_3_). These results were similar to those of other previous studies which reported on the phosphate and zinc-solubilizing abilities of both yeast species [26,65,80,81,82]. Moreover, some species of yeast in the genera Aureobasidium, Barnettozyma, Candida, Holtermanniella, Lachancea, Pichia, Rhodotrula, Saccharomyces, Torulaspora have been recognized for their phosphate and zinc-solubilizing abilities [26,29,80,81,82,83,84]. Additionally, a number of previous studies have reported that the insoluble mineral solubilization ability of yeast cultures can vary depending on the type of mineral and yeast species as well as the particular yeast strain [26,29,83,84].

The ability of yeast to produce extracellular enzymes, including amylase, endoglucanase, lipase, pectinase, protease and xylanase, was investigated in this study. It was found that the ability to produce enzymes was dependent upon the yeast species and strain, which is in accordance with the findings of several previous studies [26,28,29]. Another mechanism through which microorganisms inhibit plant pathogenic organisms is the production of cell wall-degrading enzymes. The cell wall-degrading enzymes, especially cellulase (endoglucanase, exoglucanase and β-glucosidase) and protease, can affect the structural integrity of the walls of the target pathogen, while indirectly promoting the growth of the host plant [85,86]. Some species of yeast in the genera Candida, Cryptococcus, Debaryomyces, Holtermaniella, Moesziomyces, Rhodotorula, Saccharomyces, Schwanniomyces and Trichosporon have been reported for cellulase and protease producers [26,84,87,88,89,90]. In our study, all strains of *R. ruineniae, S. pseudopolymorphus, T. asahii, T. coremiiforme* and *W. anomalus* produced endogulcanase. Notably, *P. laurentii* has been reported to produce endogulcanase [91], but none of the *P. laurentii* strains obtained in this study produced this enzyme. The protease production of *P. laurentii* and *W. anomalus* observed in this study has also been reported by previous studies [26,84,85].

Our study found that the yeast strains belonging to *P. laurentii* and *W. anomalus* displayed the most promising plant growth promoting activities. Previous studies reported that *P. laurentii* and *W. anomalus* could be isolated from soil samples, and they displayed multifarious plant growth promoting activity [26,28,69,81,91]. In 2016, Moller et al. [92] reported that the *L. laurentii* strain CAB 91 isolated from the rhizosphere soil of the blue lupin (*Lupinus angustifolius*) could enhance the growth of the blue lupin and improve its nutritional physiology. Though these species were reported as a PGPM, they are considered opportunistic human pathogens [93,94]. Therefore, the strains of PGPMs may represent a potential threat to humans, animals and the environment. Thus, more information is required regarding the toxicity of these strains, and clinical tests in the laboratory should be conducted for the purposes of safety before they are used [95,96].

## 5. Conclusions

Currently of particular relevance is the search for microorganisms that display multifunctional biological activity. In this study, yeast strains isolated from the Assam tea soil collected from plantations located in northern Thailand have shown multifarious plant growth promoting traits. Our results indicate the potential application of these yeast strains, especially *P. laurentii* and *W*. *anomalus*, in various agricultural areas and specifically for the development of biofertilizers. However, some yeast strains may represent a potential threat to humans, animals or the health of plants. Therefore, laboratory assays of their toxicity are necessary, and clinical tests are needed in future studies to fully understand the profile of these yeast strains. Important and vital research must be done on these yeast strains before their application can be approved. Additionally, field trials should be conducted in the future, particularly with regard to the management and safety of these yeast strains.

## Figures and Tables

**Table 1:** Location, soil type, pH value and electrical conductivity of collected soil samples.

Site	Location	Soil Type	pH Value	EC (µS/cm)
S1	Skad, Pua District, Nan Province	Sandy clay loam	6.13	0.048
S2	Sri Na Pan, Muang District, Nan Province	Sandy clay loam	6.06	0.046
S3	Thep Sadej, Doi Saket District, Chiang Mai Province	Sandy clay loam	6.54	0.049

**Table 2:** Identification of yeast strains obtained in this study based on analysis of the D1/D2 region of the large subunit ribosomal RNA (LSU rRNA) gene.

Site	Strain SDBR-CMU	GenBank Number	Closeted Type Species/Similarity (%)	Identification
S1	S1-01	MT613405	*Trichosporon asahii* CBS 2479/100	*T. asahii*
	S1-02	MT613406	*Papiliotrema laurentii* CBS 139 99.87	*P. laurentii*
	S1-03	MT613407	*Rhodosporidiobolus ruineniae* CBS 5001/100	*R. ruineniae*
	S1-04	MT623567	*Trichosporon asahii* CBS 2479/100	*T. asahii*
	S1-05	MT623565	*Rhodosporidiobolus ruineniae* CBS 5001/100	*R. ruineniae*
	S1-06	MT613408	*Wickerhamomyces anomalus* CBS 5759/100	*W. anomalus *
	S1-07	MT613409	*Trichosporon asahii* CBS 2479/100	*T. asahii*
	S1-08	MT623566	*Trichosporon asahii CBS 2479/100 *	*T. asahii*
	S1-09	MT623568	*Rhodosporidiobolus ruineniae* CBS 5001/100	*R. ruineniae*
	S1-10	MT613410	*Aureobasidium melanogenum* CBS 105.22/100	*A. melanogenum*
	S1-11	MT613411	*Wickerhamomyces xylosivorus* NBRC 111553/89.85	*Wickerhamomyces* sp. 1
S2	S2-01	MT613683	*Rhodosporidiobolus ruineniae* CBS 5001/100	*R. ruineniae*
	S2-02	MT623569	*Wickerhamomyces xylosivorus* NBRC 111553/89.85	*Wickerhamomyces* sp. 1
	S2-03	MT623575	*Trichosporon asahii* CBS 2479/100	*T. asahii*
	S2-04	MT613721	*Papiliotrema laurentii* CBS 139/99.87	*P. laurentii*
	S2-05	MT623576	*Rhodosporidiobolus ruineniae* CBS 5001/100	*R. ruineniae*
	S2-06	MT613722	*Wickerhamomyces xylosivorus* NBRC 111553/89.85	*Wickerhamomyces* sp. 1
	S2-07	MT623572	*Aureobasidium melanogenum* CBS 105.22/100	*A. melanogenum*
	S2-08	MT623573	*Trichosporon asahii* CBS 2479/100	*T. asahii*
	S2-09	MT613858	*Curvibasidium pallidicorallinum* CBS 9091/100	*C. pallidicorallinum*
	S2-10	MT623570	*Wickerhamomyces anomalus* CBS 5759/100	*W. anomalus *
	S2-11	MT623574	*Rhodosporidiobolus ruineniae* CBS 5001/100	*R. ruineniae*
	S2-12	MT613867	*Curvibasidium pallidicorallinum* CBS 9091/100	*C. pallidicorallinum*
	S2-13	MT613870	*Kazachstania aquatica* CBS 10102/100	*K. aquatica*
	S2-14	MT623571	*Wickerhamomyces mori* CBS 12678/90.97	*Wickerhamomyces* sp. 2
	S2-15	MT613872	*Kazachstania aquatica* CBS 10102/100	*K. aquatica*
	S2-16	MT613876	*Papiliotrema laurentii* CBS 139/99.85	*P. laurentii*
	S2-17	MT613875	*Wickerhamomyces mori* CBS 12678/90.97	*Wickerhamomyces* sp. 2
S3	S3-01	MT626064	*Schwanniomyces pseudopolymorphus* CBS:2008/100	*S. pseudopolymorphus*
	S3-02	MT626065	*Apiotrichum scarabaeorum* CBS 5601/99.84	*Ap. scarabaeorum*
	S3-03	MT632028	*Schwanniomyces pseudopolymorphus* CBS:2008/100	*S. pseudopolymorphus*
	S3-04	MT626066	*Saturnispora diversa* NRRL Y-5713/100	*Sat. diversa*
	S3-05	MT639220	*Wickerhamomyces xylosivorus* NBRC 111553/89.85	*Wickerhamomyces* sp. 1
	S3-06	MT626068	*Galactomyces pseudocandidum* CBS 820.71/97.87	*Galactomyces* sp.
	S3-07	MT632029	*Papiliotrema laurentii* CBS 139/99.86	*P. laurentii*
	S3-08	MT626069	*Trichosporon coremiiforme* CBS 2482/99.87	*T. coremiiforme*
	S3-09	MT622026	*Trichosporon asahii* CBS 2479/100	*T. asahii*
	S3-10	MT626071	*Saturnispora sekii* CBS 10931/100	*Sat. sekii*
	S3-11	MT632027	*Rhodosporidiobolus ruineniae* CBS 5001/100	*R. ruineniae*
	S3-12	MT632031	*Trichosporon coremiiforme* CBS 2482/99.87	*T. coremiiforme*
	S3-13	MT632030	*Saturnispora sekii* CBS 10931/100	*Sat. sekii*
	S3-14	MT632025	*Trichosporon asahii* CBS 2479/100	*T. asahii*

**Table 3:** Production of indole-3-acetic acid by yeast strains in this study.

Yeast Species	Strain SDBR-CMU	Amount of IAA (mg/L) *
*Trichosporon asahii*	S1-01	3.21 ± 2.19
	S1-04	3.65 ± 1.34
	S1-07	2.12 ± 1.35
	S1-08	4.21 ± 1.87
	S2-03	2.53 ± 1.46
	S2-08	5.02 ± 2.05
	S3-09	3.82 ± 2.14
	S3-14	2.35 ± 0.92
*Papiliotrema laurentii*	S1-02	2.35 ± 1.97
	S2-04	3.54 ± 1.06
	S2-16	2.78 ± 1.53
	S3-07	2.93 ± 2.04
*Rhodosporidiobolus ruineniae*	S1-03	37.32 ± 3.05
	S1-05	20.45 ± 2.67
	S1-09	18.38 ± 1.28
	S2-01	26.73 ± 3.24
	S2-05	17.45 ± 2.03
	S2-11	14.63 ± 2.15
	S3-11	23.45 ± 3.04
*Wickerhamomyces* sp. 1	S1-11	17.36 ± 2.12
	S2-02	12.45 ± 3.16
	S2-06	10.38 ± 2/18
	S3-05	16.63 ± 1.96
*Curvibasidium pallidicorallinum*	S2-09	8.61 ± 1.37
	S2-12	3.62 ± 1.75
*Apiotrichum scarabaeorum*	S3-02	4.24 ± 1.48
*Trichosporon coremiiforme*	S3-08	4.56 ± 2.42
	S3-12	3.52 ± 1.87

* Data are means ± standard deviation of three replicates.

**Table 4:** Solubilization of insoluble minerals by yeast strains in this study.

Yeast Species	Strain SDBR-CMU	Solubilization Index */Solubilization Activities		
		Ca_3_(PO_4_)_2_	ZnO	ZnCO_3_
*Papiliotrema laurentii*	S1-02	1.32 ± 0.94/Medium	2.13 ± 0.92/High	–
	S2-04	1.04 ± 0.54/Medium	2.05 ± 0.32/High	–
	S2-16	1.26 ± 0.72/Medium	2.08 ± 0.24/High	–
	S3-07	1.26 ± 0.36/Medium	2.07 ± 0.36/High	–
*Wickerhamomyces anomalus*	S1-06	1.56 ± 0.61/Medium	1.34 ± 0.21/Medium	1.52 ± 0.31/Medium
	S2-10	1.48 ± 0.17/Medium	1.27 ± 0.35/Medium	1.38 ± 0.52/Medium

* Data are means ± standard deviation of five replicates. “–” = non-solubilization.

**Table 5:** Enzyme production of yeast strains in this study.

Yeast Species	Strain SDBR-CMU	Enzyme Activity Index *					
		Amylase	Endoglucanase	Lipase	Pectinase	Protease	Xylanase
*Trichosporon asahii*	S1-01	–	1.57	1.25	–	–	–
	S1-04	–	1.43	1.28	–	–	–
	S1-07	–	1.03	1.27	–	–	–
	S1-08	–	1.34	1.17	–	–	–
	S2-03	–	1.06	1.24	–	–	–
	S2-08	–	1.13	1.07	–	–	–
	S3-09	–	1.15	1.04	–	–	–
	S3-14	–	1.23	1.08	–	–	–
*Papiliotrema laurentii*	S1-02	1.01	–	1.30	1.63	1.04	1.01
	S2-04	1.13	–	1.21	1.54	1.09	1.02
	S2-16	1.08	–	1.33	1.46	1.12	1.03
	S3-07	1.05	–	1.08	1.38	1.07	1.06
*Rhodosporidiobolus ruineniae*	S1-03	–	1.07	1.09	–	–	1.01
	S1-05	–	1.04	1.09	–	–	1.06
	S1-09	–	1.34	1.07	–	–	1.04
	S2-01	–	1.12	1.37	–	–	1.02
	S2-05	–	1.13	1.07	–	–	1.08
	S2-11	–	1.09	1.07	–	–	1.13
	S3-11	–	1.04	1.10	–	–	1.03
*Apiotrichum scarabaeorum*	S3-02	–	–	–	–	–	1.04
*Trichosporon coremiiforme*	S3-08	–	1.07	1.13	–	–	–
	S3-12	–	1.05	1.32	–	–	–
*Wickerhamomyces anomalus*	S1-06	–	1.01	–	–	1.03	–
	S2-10	–	1.06	–	–	1.24	–
*Kazachstania aquatica*	S2-13	1.18	1.37	–	–	–	–
	S2-15	1.12	1.32	–	–	–	–
*Schwanniomyces pseudopolymorphus*	S3-01	1.05	1.16	–	–	–	–
	S3-03	1.04	1.12	–	–	–	–
*Aureobasidium melanogenum*	S1-10	–	–	–	–	–	1.07
	S2-07	–	–	–	–	–	1.05
*Galactomyces sp.*	S3-06	–	–	–	–	–	1.01
*Saturnispora diversa*	S3-04	–	–	1.20	–	–	–

* Data are means of five replicates. “–” = non-production.
